# Subclass-specific IgG glycosylation is associated with markers of inflammation and metabolic health

**DOI:** 10.1038/s41598-017-12495-0

**Published:** 2017-09-26

**Authors:** Rosina Plomp, L. Renee Ruhaak, Hae-Won Uh, Karli R. Reiding, Maurice Selman, Jeanine J. Houwing-Duistermaat, P. Eline Slagboom, Marian Beekman, Manfred Wuhrer

**Affiliations:** 1 Center for Proteomics and Metabolomics, Leiden University Medical Center, Leiden, The Netherlands; 2 Department of Clinical Chemistry and Laboratory Medicine, Leiden University Medical Center, Leiden, The Netherlands; 3 Department of Medical Statistics and Bioinformatics, Leiden University Medical Center, Leiden, The Netherlands; 4 Department of Molecular Epidemiology, Leiden University Medical Center, Leiden, The Netherlands; 5 Present Address: Pharming Group N.V., Leiden, The Netherlands

## Abstract

This study indicates that glycosylation of immunoglobulin G, the most abundant antibody in human blood, may convey useful information with regard to inflammation and metabolic health. IgG occurs in the form of different subclasses, of which the effector functions show significant variation. Our method provides subclass-specific IgG glycosylation profiling, while previous large-scale studies neglected to measure IgG2-specific glycosylation. We analysed the plasma Fc glycosylation profiles of IgG1, IgG2 and IgG4 in a cohort of 1826 individuals by liquid chromatography-mass spectrometry. For all subclasses, a low level of galactosylation and sialylation and a high degree of core fucosylation associated with poor metabolic health, i.e. increased inflammation as assessed by C-reactive protein, low serum high-density lipoprotein cholesterol and high triglycerides, which are all known to indicate increased risk of cardiovascular disease. IgG2 consistently showed weaker associations of its galactosylation and sialylation with the metabolic markers, compared to IgG1 and IgG4, while the direction of the associations were overall similar for the different IgG subclasses. These findings demonstrate the potential of IgG glycosylation as a biomarker for inflammation and metabolic health, and further research is required to determine the additive value of IgG glycosylation on top of biomarkers which are currently used.

## Introduction

Glycosylation is known to reflect the physiological state of an organism and changes thereof^1^. For immunoglobulin G (IgG), which occupies a central role in the immune system, it is known that the conserved *N*-glycan located at asparagine 297 on the fragment crystallisable (Fc) part of IgG can modulate inflammatory responses: a lack of core fucose, galactose and *N*-acetylneuraminic (sialic) acid increases the ability of IgG to induce antibody-dependent cell-mediated cytotoxicity (ADCC) in mice^2–4^. Furthermore, glycosylation of IgG is associated with various pathologies. Autoimmune diseases are generally associated with decreased galactosylation and sialylation of IgG Fc, which is a hallmark of the inflammatory state of these pathologies^5–10^. Different types of cancer^11–13^ and viral infections^14,15^ have been shown to exhibit low galactosylation and sialylation of IgG *N*-glycans similar to inflammatory conditions. IgG glycosylation, specifically low galactosylation and sialylation, has also been proposed as a biomarker of inflammageing, the state of chronic weak inflammation in elderly individuals^16^, or more generally as a marker of immune activation^17^.

Since IgG glycosylation is known to be altered during a state of inflammation, it is not surprising that associations between certain glycoforms and the inflammatory marker C-reactive protein (CRP) have been reported^18–21^. Inflammation often goes hand in hand with ageing and poor metabolic health, which are all associated with a higher risk for cardiovascular disease^22^. However, an overview is lacking of the associations between IgG glycosylation and an assortment of clinical markers related to metabolic health in healthy individuals. Furthermore, other large cohort studies (>100 healthy participants) on IgG glycosylation report either a released glycan profile^23,24^, which does not contain any subclass-specific information, or a joint profile for IgG2 and IgG3^25,26^. This is due to the shared peptide sequence of the tryptic glycopeptides of these two subclasses, which prevents separate analysis by mass spectrometry, and thereby prevents separate examination of IgG2 glycosylation. From *in vitro* assays it is known that IgG2 exhibits a lower overall binding affinity to Fc gamma receptors (FcγRs)^27–29^ and ADCC capacity^30,31^.

In order to evaluate the merit of IgG Fc *N*-glycosylation as a systemic biomarker of metabolic health, we here analysed glycosylation of IgG1, 2 and 4 in a cohort of 1826 healthy individuals to investigate associations with known markers related to inflammation and metabolic health. The population analysed in this study is part of the Leiden Longevity Study, aimed at determining the biological foundation of healthy ageing. In this study we investigate the glycosylation of IgG in relation to measurements of inflammation, such as plasma levels of C-reactive protein (CRP) and interleukin-6 (IL-6), and of metabolism, such as lipids and thyroid hormone. IgG glycosylation analysis has previously been performed on this cohort with MALDI-MS to facilitate the measurement of 6 *N*-glycopeptides for IgG1 and IgG2 each, allowing only the assessment of galactosylation and bisection^18^. The current analysis is performed with nanoLC-ESI-QTOF-MS, which offers enhanced sensitivity and a more extensive glycoprofiling of the samples: 20 *N*-glycopeptides were identified for IgG1 and IgG2 and 10 for IgG4, providing novel information on the sialylation and fucosylation of these samples.

## Methods

### Cohort participants and study design

This study was performed on 1995 available plasma samples from 1170 offspring of nonagenarian siblings of the Leiden Longevity cohort and 656 of their partners as controls^32^. Families were included if the parents were over 91 (for females) or over 89 (for males) years of age. Previously we have shown that comparison of the middle-aged offspring of long-lived parents and their partners as controls reveals parameters associating with familial metabolic health, thereby demonstrating that offspring have a beneficial immune-metabolic health^33–35^. An overview of the age and sex distribution of the participants can be seen in Table 1. The prevalence of type 2 diabetes and myocardial infarction was 5.2% and 2.9%, respectively, and prescriptions for lipid-lowering medication and anti-hypertensives were being taken by 11.3% and 21.7% of the individuals in our sample set^36^. Venous blood was collected under non-fasting conditions. All participants have given informed consent prior to sample collection, in accordance with the Declaration of Helsinki. The study design and protocols were approved by the Ethical Committee of the Leiden University Medical Center. All experiments were carried out in accordance with relevant guidelines and regulations.

**Table 1:** Distribution of age, sex, glycosylation features and metabolic parameters within the Leiden Longevity study population.

	mean	SD	range	N
**Age (years)**	59.1	+/−6.7	30.2–79.2	1826
**Sex**	47.1% male	—	—	1826
**CRP (mg/L)**	2.86	+/−9.5	0.2–228.7	1813
**IL-6 (pg/mL)**	0.67	+/−1.2	0–19.6	1705
**Glucose (mmol/L)**	5.94	+/−1.5	2.5–26.3	1816
**Insulin (mU/L)**	23.23	+/−22.1	2–238	1766
**TC (mmol/L)**	5.55	+/−1.2	1.4–10.8	1820
**LDLC (mmol/L)**	3.33	+/−1.0	0.7–7.9	1772
**HDLC (mmol/L)**	1.42	+/−0.4	0.2–3.2	1819
**TG (mmol/L)**	1.82	+/−1.2	0.2–21.2	1820
**free T3 (pmol/L)**	4.12	+/−0.8	1.8–14.3	1819
**smoking**	13.6% smoking	—	—	1587
**CMV serostatus**	45.9% positive	—	—	1437
**longevity**	64.1% offspring	—	—	1826
**IgG1 fucosylation**	91.2%	+/−4.1	69.3–99.5	1825
**IgG2 fucosylation**	97.4%	+/−0.9	92.6–99.1	1826
**IgG1 bisection**	19.1%	+/−3.3	6.5–35.8	1825
**IgG2 bisection**	13.9%	+/−2.7	6.4–26.5	1826
**IgG4 bisection**	19.6%	+/−4.4	8.8–41.3	1742
**IgG1 galactosylation**	50.1%	+/−6.4	24.6–70.8	1825
**IgG2 galactosylation**	39.5%	+/−6.2	9.0–61.3	1826
**IgG4 galactosylation**	43.7%	+/−6.9	14.5–66.0	1742
**IgG1 sialylation**	6.8%	+/−1.4	2.5–14.0	1825
**IgG2 sialylation**	6.2%	+/−1.4	1.4–11.9	1826
**IgG4 sialylation**	9.1%	+/−2.0	3.2–18.9	1742
**IgG1 sialic acid per gal**	13.6%	+/−1.6	9.0–21.5	1825
**IgG2 sialic acid per gal**	15.5%	+/−1.7	9.4–22.5	1826
**IgG4 sialic acid per gal**	20.7%	+/−2.0	13.8–30.8	1742

The mean, standard deviation (SD), range (minimum and maximum value) and number of included samples (N) are given. Samples that were excluded based on data quality criteria were not used for calculation of values in this table.

### Metabolic parameter analysis

Various parameters related to metabolic health were determined for the participants of the Leiden Longevity cohort. High-sensitivity measurements of CRP, glucose, total cholesterol (TC), high-density lipoprotein cholesterol (HDLC) and triglycerides (TG) in blood samples were performed on the Hitachi Modular P-800, while free triiodothyronine (T3) levels were assessed using a Modular E-170 (both Roche Diagnostics, Almere, the Netherlands)^37–39^. Low-density lipoprotein cholesterol (LDLC) was derived from these measurements using the Friedewald formula (LDLC = TC − HDLC − TG/5)^40^. If the level of TG exceeded 4.52 mmol/L, LDLC was listed as missing. The level of insulin in blood was assessed with an Immulite 2500 (DPC, Los Angeles, CA)^39^. Interleukin 6 (IL-6) levels were determined with an enzyme-linked immunosorbent assay (ELISA) (Sanquin Reagents, Amsterdam, The Netherlands). Furthermore, the self-reported current smoking status of the participants was registered. An ELISA was done on serum to determine if the participants had antibodies against cytomegalovirus (PKS Assay, Medac, Wedel, Germany). The distributions of these metabolic parameters within the cohort are listed in Table 1.

### IgG glycopeptide sample preparation

IgGs were purified in 96-well format as described previously^18^. An amount of 2 µL of plasma was incubated with 15 µL Protein A Sepharose Fast Flow beads (GE Healthcare, Uppsala, Sweden) and 150 µL phosphate buffered saline (PBS) on a 96-well filter plate for 1 hour at room temperature while shaking. The IgG samples were washed three times with PBS and three times with MilliQ-purified water, followed by elution with 100 µL 100 mM formic acid. The samples were then dried in a vacuum concentrator for 2 hours at 60 °C and resuspended in 40 µL 25 mM ammonium bicarbonate with 200 ng of trypsin (sequencing grade modified trypsin, Promega, Madison, WI). Digestion took place overnight at 37 °C. Two of the 96-well plates were prepared twice to assess interbatch variation.

### NanoLC-ESI-QTOF-MS analysis

The IgG glycopeptide samples were analysed using liquid chromatography coupled to mass spectrometry (LC-MS), in a setup described previously^41^. An amount of 2.5 µL of the samples was injected in an Ultimate 3000 RSLCnano liquid chromatography system (Dionex, Sunnyvale, CA). The samples were first washed on an Acclaim PepMap100 C18 trap column (5 mm × 300 µm i.d., Dionex, Sunnyvale, CA), and subsequently separated on an Ascentis Express C18 nanoLC column (50 mm × 75 µm i.d., 2.7 µm HALO fused core particles; Supelco, Bellefonte, PA) with a flow rate of 0.9 µL/min. The following linear gradient was used, with solvent A consisting of 0.1% trifluoroacetic acid and B of 95% acetonitrile (ACN): t = 0, 3% solvent B; t = 2, 6%; t = 4.5, 18%; t = 5, 30%; t = 7, 30%; t = 8, 0%; t = 11, 0%.

Via a sheath-flow electrospray (ESI) interface (Agilent Technologies, Santa Clara, CA), the LC was coupled to a Maxis Impact quadrupole time-of-flight (QTOF)-MS system (micrOTOF-Q; Bruker Daltonics, Bremen, Germany). A sheath-flow consisting of 50% isopropanol, 20% propionic acid (Merck) and 30% MilliQ-purified water was applied at 2 µL/min, and nitrogen gas was applied at 4 L/min. MS1 spectra were acquired with a frequency of 0.5 Hz and within an *m/z* range of 600-2000. An IgG standard and two blank injections were run in between every 12 runs.

### Glycosylation data processing

Glycosylation profiles were extracted from the data files as described previously^41^. The three subclass-specific types of tryptic glycopeptides (IgG1: EEQYNSTYR, IgG2: EEQFNSTFR, IgG4: EEQFNSTYR) eluted at different time points, with the retention times of differentially glycosylated variants of each subclass clustered closely together, since the secondary interaction between sialic acids and the silica column which usually leads to later elution of sialylated glycopeptides was negated by the use of TFA in the mobile phase (Supplemental Figure S1). Based on their mass and previous characterizations of IgG *N*-glycan structures^42–44^, 20 *N*-glycans were identified for both IgG1 and IgG2 (Supplemental Table S1). For IgG4, only 10 glycopeptides could be identified, as the mass of the minor afucosylated species overlapped with the much more abundant IgG1 fucosylated glycopeptides, and could thus not be reliably distinguished from tailing of the latter (Supplemental Table S1).

LC-MS data was examined and calibrated in Compass Data Analysis 4.2 (Bruker Daltonics), based on a list of four of the most abundant *N*-glycopeptides (G0F, G1F, G2F, G2FS) in both double and triple charge state. The data was then exported in mzXML file format. Alignment of the retention times was done using MSalign. Next, the in-house developed software tool Xtractor 2D^45^ was used to extract the signal intensity of the first three isotopic peaks of various glycopeptides in both double and triple charge state, within an *m/z* window of ±0.04 Thompson and a time window of ±10 s surrounding the retention time. These *N*-glycopeptides were inputted from a list of *m/z* values (Supplemental Table S1) encompassing 20 IgG1 glycopeptides, 20 IgG2 glycopeptides and 10 IgG4 glycopeptides (afucosylated IgG4 species overlapped with IgG1 glycopeptides and thus could not be properly analysed).

For each *N*-glycopeptide, the signal intensity of the three isotopic peaks in double and triple charge state were background-corrected and summed. IgG2-G1FNS1 (the IgG2 glycan carrying 1 galactose (G1), 1 sialic acid (S1), a core fucose (F) and a bisecting N-acetylglucosamine) and IgG2-G2FNS1, which each represented less than 0.4% of the total IgG2 glycopeptide abundance, were excluded from the data due to a severe batch effect. The *N*-glycopeptide signals were normalised by dividing each by the total signal intensity of all *N*-glycopeptides belonging to that subclass, yielding percentage data amounting to 100% per subclass.

An intensity threshold was determined based on the percentage of the signal intensity which was derived from *N*-glycopeptides with a signal-to-background ratio larger than 3. If the total intensity per subclass did not exceed the threshold, data from that subclass was excluded. After this exclusion, IgG1 data remained for 1825 individuals, IgG2 for 1826 and IgG4 for 1742.

### Data preprocessing

From the individual *N*-glycan percentage data, five glycosylation features were determined for each IgG subclass (calculations can be found in Supplemental Table S2): fucosylation (% of fucosylated *N*-glycopeptides), bisection (% of *N*-glycopeptides carrying a bisecting *N*-acetylglucosamine (GlcNAc)), galactosylation (% of galactoses per antennae), sialylation (% of sialic acids per antennae) and sialic acid per galactose (% of sialic acids per galactose). Fucosylation could not be determined for IgG4, since no afucosylated IgG4 glycopeptides were measured due to overlap with IgG1 glycopeptides.

The three most abundant glycopeptides (G0F, G1F, G2F) of the standard IgG samples run in between every twelve samples showed a relative standard deviation (RSD) of 4.7%. The average intraplate RSD was 1.9%, based on standard IgG samples run during the measurement of each plate, and the interplate RSD was 3.3%. Therefore, batch correction was done using the ComBat package^46^ in R 3.0.1, with the 96-well plates as batches^47^. Furthermore, any glycosylation feature value which deviated more than 5 standard deviations (SD) from the mean was replaced by 5 × SD from the mean.

To not violate the assumption of linear regression that variables are normally distributed, we transformed the glycosylation features and several of the metabolic parameters (CRP, IL-6, glucose, insulin, TG, free T3) using a natural logarithm.

### Reproducibility of the analysis

Two 96-well plates with samples were purified and analysed twice, to assess the reproducibility of our method. To assess the level of correlation between these sample plates, a Pearson’s correlation test was performed in R using the function cor.test(). The glycosylation features on the different plates showed a high level of correlation, i.e. Pearson’s correlation coefficients (r) of 0.97 (fucosylation), 0.95 (bisection), 0.98 (galactosylation), 0.93 (sialylation) and 0.80 (SA per gal) for IgG1. From the two plates which were prepared twice, the plate which showed the highest signals was used for further data analysis.

### Statistical association analysis

Statistical analyses were done in R 3.0.1^47^. A paired t-test from the R stats package was performed to see if there was a significant difference in glycosylation features between different subclasses. Regression analysis was done using the generalised estimating equation package (geepack)^48^ to account for family relationship. A linear model with formula ‘glycosylation feature Y ~ β_1_*metabolic parameter X + β_2_*age + β_3_*sex + β_4_*(age*sex) + error’ was fitted to the data, to assess the association between each glycosylation feature and metabolic parameter, while correcting for confounders, taking into account within-family relatedness and under the assumption of an exchangeable correlation structure. The output of this regression analysis consisted of a *p*_1_-value, β_1_ coefficient and its standard error (SE(β_1_)) describing the association between each glycosylation feature and metabolic parameter, and from this the t statistic was derived using t = β_1_/SE(β_1_). To assess the relation of IgG glycosylation with familial metabolic health, a logistic regression model was applied using the same software, with familial metabolic health as dependent variable Y, coded as 0 for controls and 1 for members of long-lived families, and IgG glycosylation features as independent variable X, while correcting for the same confounders and within-family relatedness, and under the assumption of an independent correlation structure.

Significance was defined as a *p*-value below the Bonferroni-corrected threshold α = 0.000714 (7.14 × 10^−4^). This threshold was derived from the standard threshold α = 0.05 divided by 70, which is the number of analyses between 14 variables (metabolic parameters + age + sex) and 5 glycosylation features (subclass-specific versions of the same glycosylation feature were disregarded since they are highly intercorrelated).

### Data visualization

Before plotting, glycosylation data was corrected for either age-specific differences or for both age- and sex-specific differences, by fitting the linear model (geepack) ‘glycosylation feature ~ age’ or ‘glycosylation feature ~ age + sex’, and then taking the residuals. The ggplot2 package in R was used for visualization of the data^49^. A linear trendline was fitted to plots with the geom_smooth function. A heatmap was generated in R with the weighted correlation network analysis (WGCNA) package^50^.

## Results

Tryptic *N*-glycopeptides of IgG2 and IgG3 share the same peptide sequence in Caucasians^51,52^, and so the commonly used protein G enrichment of IgG gives a joint profile for both IgG2 and IgG3. In this study we use Protein A for IgG purification, which has been shown to capture IgG1, 2 and 4, but has a much lower binding affinity for IgG3 under the enrichment conditions we use^53^, thus allowing for near-separate glycoprofiling of IgG2.

By performing nanoLC-ESI-QTOF-MS analysis on tryptic IgG glycopeptides, Fc glycosylation profiles of IgG1, IgG2 and IgG4 were determined for nearly 2000 participants of the Leiden Longevity study. After data curation, IgG1, IgG2 and IgG4 data remained for respectively 1825, 1826 and 1742 participants. From the individual *N*-glycan data five glycosylation features were derived (the calculations can be found in Supplemental Table S2): fucosylation, bisection, galactosylation, sialylation and sialic acid per galactose (SA per gal). While the type of sialic acid linkage could not be determined by our methods, it is known from literature that the vast majority of sialic acids from plasma-derived Fc-IgG are 2,6-linked^54–56^.

The distribution of the age and sex of the study population, as well as the average measurements of metabolic parameters and IgG glycosylation features, can be found in Table 1. IgG glycosylation was observed to be age-dependent and differences were seen between males and females (Supplemental Figure S2), as has been described in earlier reports^26,57–59^. We observe significant differences between the glycosylation features of the three IgG subclasses: t-tests revealed a *p*-value below 1.0 × 10^−10^ for all comparisons of glycosylation features between IgG subclasses (1 vs 2, 1 vs 4 and 2 vs 4). IgG2 exhibits a higher level of fucosylation (IgG1: 91.2 ± 4.1% (mean ± SD); IgG2: 97.4 ± 0.9%; IgG4 fucosylation could not be determined), but a lower level of bisection (IgG1: 19.1 ± 3.3%, IgG2: 13.9 ± 2.7%, IgG4: 19.6 ± 4.4%) and galactosylation (IgG1: 50.1 ± 6.4%, IgG2: 39.5 ± 6.2%, IgG4: 43.7 ± 6.9%) as well as sialylation (IgG1: 6.8 ± 1.4%, IgG2: 6.2 ± 1.4%, IgG4: 9.1 ± 2.0%). It should be noted that galactosylation and sialylation here are defined as the percentage of galactoses/sialic acids per antenna. If defined instead as the percentage of galactoses/sialic acids per *N*-glycan, the percentages of *N*-glycans carrying at least one galactose would lie at 72.3 ± 7.0% (IgG1), 59.9 ± 7.6% (IgG2), and 62.6 ± 8.1% (IgG4), while the percentage of *N*-glyans carrying at least one sialic acid consists of 13.7 ± 2.9% (IgG1), 12.3 ± 2.7% (IgG2), and 18.0 ± 3.9% (IgG4).

### Association between IgG glycosylation and immune-metabolic parameters

**Figure 1:**
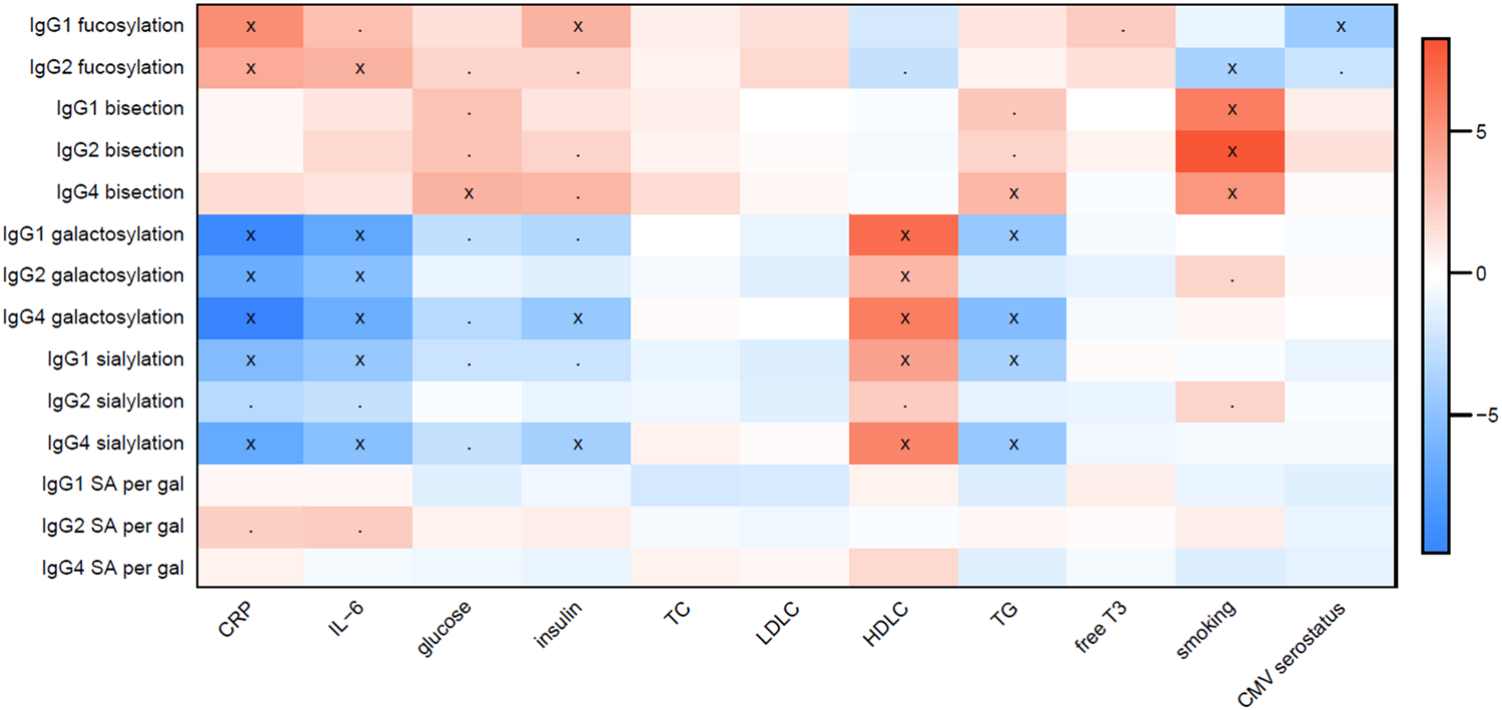
Associations between glycosylation features (y-axis) and metabolic parameters (x-axis). The colors indicate the magnitude of the association as represented by the t statistic (t_1_ = β_1_/SE(β_1_)), with positive associations shown in red and negative in blue. Associations with a *p*_1_-value below 0.05 are marked with a point, while associations with a *p*_1_-value below 7.14 × 10^−4^, which are significant after Bonferroni correction, are marked with an X.

To investigate the relationship between IgG glycosylation and metabolic health parameters, we performed regression analysis. The resulting associations between IgG glycosylation features and metabolic parameters are visualised in a heatmap (Fig. 1, Supplemental Table S3).

#### Inflammation markers

**Figure 2:**
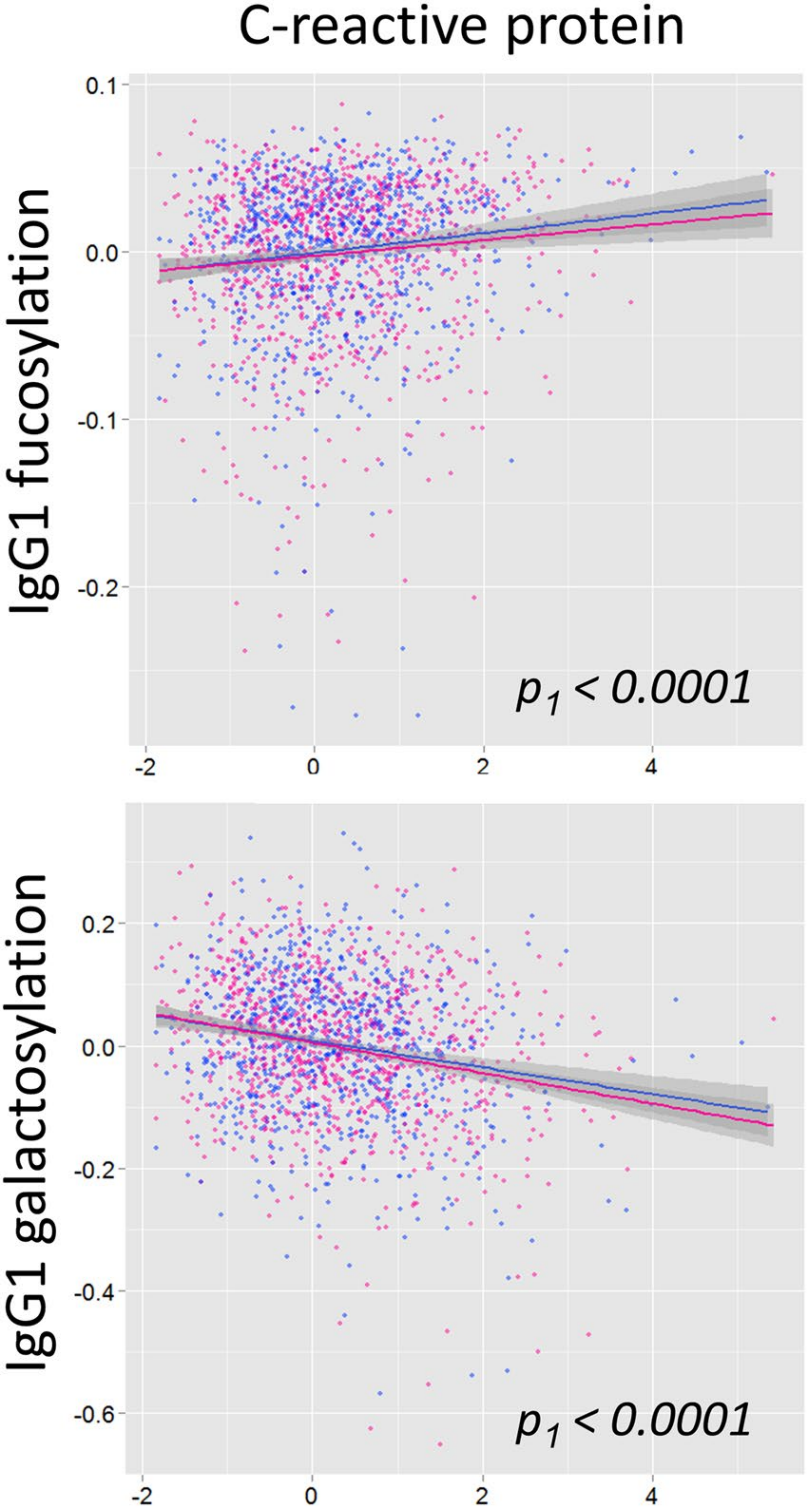
IgG1 galactosylation and fucosylation are associated with CRP. Both parameters were log transformed, and the glycosylation features were subsequently adjusted for age. Males are shown in blue and females in pink. A trend line is shown with a 95% confidence interval. *P*_*1*_-values are shown for the total population (males and females).

Low galactosylation and high fucosylation of IgG were associated with low-grade inflammation, a state of chronic inflammation characterized by elevated levels of inflammation markers CRP and IL-6, which is associated with morbidity and mortality in elderly people^60,61^ (Fig. 2) (*p*_1_ < 1.0 × 10^−4^ for all but IgG1 fucosylation, for which *p*_1_ = 2.0 × 10^−3^). IgG sialylation was also found to be significantly increased at higher levels of CRP and IL-6 (*p*_1,IgG1_ < 1.0 × 10^−4^), but sialic acid per galactose was not (*p*_1,IgG1_ = 6.2 × 10^−1^, *p*_1,IgG2_ = 1.9 × 10^−2^ for CRP). Notably, CRP only has a small contribution to the variance of IgG galactosylation (R^2^ = 0.0569 with a linear model for IgG1).

#### Metabolic markers

An increase in IgG galactosylation and sialylation (*p*_1,IgG1_ < 1.0 × 10^−4^), as well as a non-significant decrease in fucosylation (*p*_1,IgG1_ = 5.2 × 10^−2^; *p*_1,IgG2_ = 1.3 × 10^−2^) was observed at high levels of HDLC and low levels of triglycerides (TG), which together form beneficial risk profiles for cardiovascular disease, type 2 diabetes and obesity^62,63^. A negative association of galactosylation and a positive association of fucosylation were seen for insulin and glucose, which are markers for diabetes type 2 and are also known to associate with inflammation^64^. Interestingly, IgG bisection was increased at high levels of glucose and insulin (*p*_1,IgG4_ = 3.1 × 10^−4^ for glucose). Total cholesterol (TC) and LDLC did not significantly influence IgG glycosylation.

#### CMV and smoking

**Figure 3:**
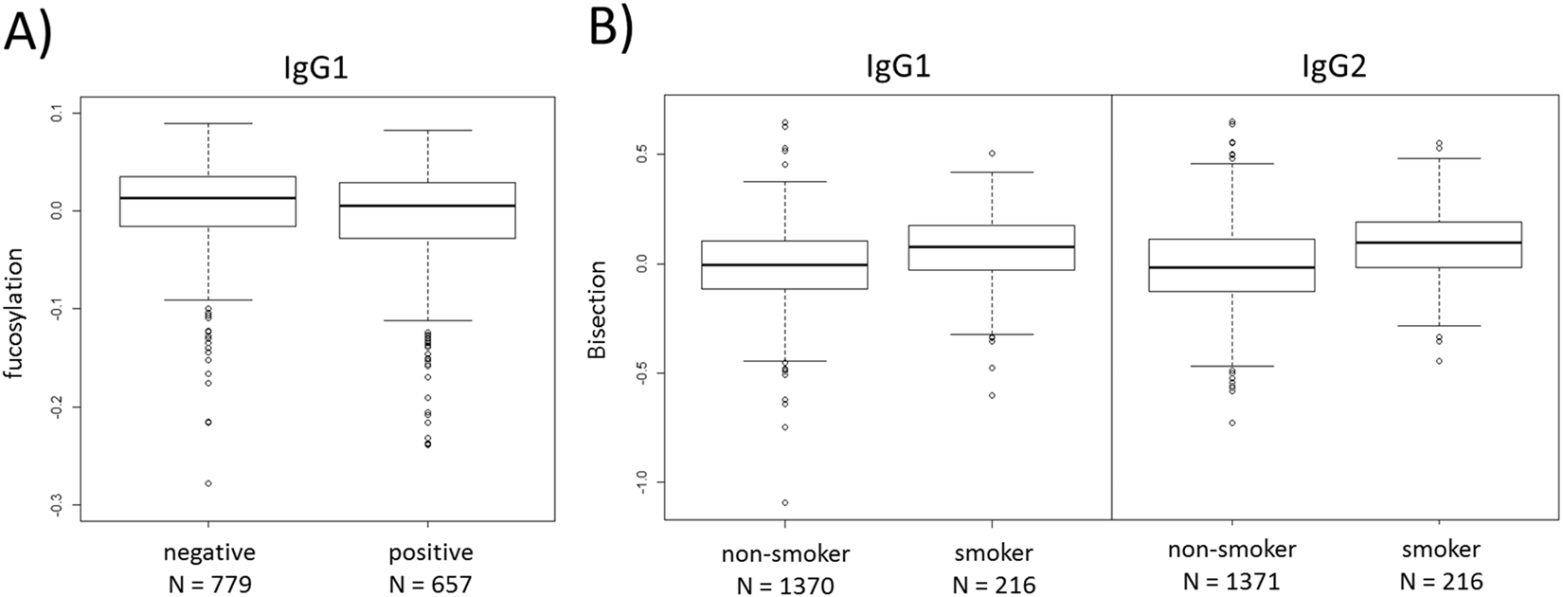
(**A**) IgG1 fucosylation of individuals with and without cytomegalovirus infection. (**B**) IgG1 and IgG2 bisection of smokers/non-smokers. The interquartile range is shown. Glycosylation data was log transformed and adjusted for age and sex.

The presence of antibodies against cytomegalovirus (CMV), a common herpes virus which remains latent in many individuals after infection, was found to be associated with low levels of IgG fucosylation. Fucosylation generally shows little variation between individuals (91.2 ± 4.1% for IgG1), so while the difference in IgG1 fucosylation between seronegative and seropositive individuals was minor (91.7 ± 3.7% versus 90.6 ± 4.3%), it was nonetheless statistically significant (*p*_1,IgG1_ < 1.0 × 10^−4^, Fig. 3A). The other glycosylation features did not show any association with CMV serostatus.

Furthermore, smokers showed slightly higher levels of bisection compared to non-smokers (*p*_1_ < 1.0 × 10^−4^ for all IgG subclasses, Fig. 3B), as well as a lower level of IgG2 fucosylation. Individuals who smoked at the time of sample collection showed a mean IgG1 bisection of 20.1 ± 3.4% versus 18.9 ± 3.2% in non-smokers.

While these differences appear to be too minor to have a significant biological influence, they might point towards larger differences in subpopulations of IgG, as is theorized in the Discussion.

### Familial metabolic health

Since we observed various associations between IgG glycosylation and parameters related to metabolic health, we were interested to know whether metabolically healthier middle-aged offspring from nonagenarians differed from controls. None of the glycosylation features showed, however, a significant difference between these groups (Supplemental Table S4, all *p*_*1*_-values > 0.02).

## Discussion

In the current study we were able to measure IgG2-specific glycosylation features, in addition to those of IgG1 and IgG4. IgG2-specific glycosylation features showed similar associations with inflammation and metabolic health markers compared to IgG1 and 4, though IgG2 glycosylation levels showed in general weaker associations for galactosylation and sialylation. Low levels of galactosylation and sialylation, as well as a high degree of fucosylation, appear to reflect an inflammatory state, poor metabolic health and potentially cardiovascular disease risk. In contrast, sialic acid per galactose was not associated with any of the metabolic markers. Furthermore, fucosylation is decreased in individuals infected with cytomegalovirus, reflecting a low-grade inflammatory state, and current smokers show higher bisecting GlcNAc levels.

### IgG subclass-specific glycosylation differences

The IgG subclasses display subtle differences in their Fc glycosylation, with IgG2 exhibiting a higher degree of fucosylation and a lower level of bisection and galactosylation compared to the other subclasses, confirming observations from a previous study which examined only four blood samples^53^. In addition, we find that galactosylation and sialylation of IgG2 generally display a weaker association (i.e. lower β coefficient) with markers of inflammation and metabolic health compared to IgG1 and IgG4. This corresponds with literature, in which it is described that IgG2 exhibits the lowest overall affinity for FcγRs^27–29^ and has the lowest ADCC capacity^30^ compared to other IgG subclasses. One may speculate that the lower capacity of IgG2 to trigger inflammatory responses, either pro-inflammatory via FcγRI, IIa, IIc, and IIIa, or anti-inflammatory via FcγRIIb, results in a weaker association with inflammation and metabolic health as compared to the other subclasses. Furthermore, it has been reported that IgG2 antibody production is primarily triggered during T-cell independent immune reactions^28,31^, which would also contribute to the weaker association with T-cell-secreted IL-6 and IL-6-stimulated CRP. Together this points towards a more limited role of IgG2 with regard to inflammation compared to the other IgG subclasses.

### Galactosylation and sialylation

Low galactosylation and sialylation are associated with a state of inflammation, which is in concordance with previous work based on MALDI-MS measurements within the same cohort^18,65^ and in children^17^. Our observations also agree with observations of decreased galactosylation and sialylation in autoimmune diseases^5–10,66–68^ and with several experimental findings that IgG without galactose and/or sialic acid has a higher inflammatory capacity^3,4,69^.

Levels of sialylation and galactosylation are highly correlated, reflecting the fact that the attachment of a sialic acid requires the presence of a galactose. We find that galactosylation and sialylation exhibit very similar associations with metabolic parameters, but the association with sialylation is generally slightly weaker than with galactosylation. The presence of sialic acid on IgG *N*-glycans is thought to confer an anti-inflammatory effect^3,69^. However, we did not observe any significant association with sialic acid per galactose, i.e. the percentage of galactoses which carry a sialic acid, indicating that associations with sialylation may be mediated by its close correlation with galactosylation. This is in line with several previous studies which concluded that galactosylation of human IgG, rather than sialylation, appears to show a pronounced negative association with clinical parameters and infection^5,70^. Together, this supports the notion that IgG Fc galactosylation may be involved in modulating inflammation.

### Fucosylation

We find that IgG fucosylation is increased in individuals with a higher level of inflammation. Despite the fact that functional studies have shown that the lack of a core fucose in IgG glycosylation greatly increases the inflammatory capacity through increased binding to FcγRIIIa^71–74^, an increase in IgG fucosylation is sometimes observed in autoimmune patients^66–68^. Furthermore, a previous study using UHPLC found that high CRP levels were associated with high fucosylation in non-galactosylated IgG *N*-glycans^75^, further demonstrating that IgG fucosylation is increased during a state of inflammation.

In individuals who tested seropositive for cytomegalovirus (CMV), a common virus which can remain in the body in a latent state after infection, a slightly lower level of fucosylation of IgG was observed than in non-infected individuals. While the differences in fucosylation are minor, it should be noted that these are individuals with a latent, asymptomatic CMV infection, and it would be interesting to investigate if a larger decrease in fucosylation is seen during active disease. Furthermore, the changes in fucosylation of total IgG may reflect a larger shift towards afucosylation in the subpopulation of anti-CMV IgG. Low IgG fucosylation has also been observed in other antigen-specific IgG populations – in alloantibody reactions against platelets and red blood cells during pregnancy or blood transfusion (as low as 12%)^76^ and in anti-HIV antibodies (~75%) – and has been suggested to be a defense mechanism by B-cells to increase antiviral control^77^. It may be interesting to investigate autoantibodies against CMV, as well as other types of infectious agents, to determine whether low fucosylation of IgG might be a hallmark of viral infections.

### Associations with longevity and metabolic health

Though we find that the IgG glycosylation reflects poor metabolic health, we were unable to find significant differences between middle-aged members of long-lived families and controls, who are known to differ on various metabolic measures^33,36,38^. Previous work which applied MALDI-MS to the same set of samples reported a significant decrease in the level of bisection of offspring of long-lived people, but only in individuals below 60 years of age^18^, and IgG glycosylation features contributed to the differentiation of controls and members of long-lived families^78^. A recent investigation into the released *N*-glycan profile of plasma samples within the Leiden Longevity cohort also could not find any association with longevity, while replicating several of the associations we find with *N*-glycans which likely originate from IgG^65^.Our study design does not allow for estimation of whether IgG glycosylation features offer a predictive value for cardiovascular disease in addition to traditional markers of inflammation. However, we did test whether IgG parameters associate with metabolic health markers and which of the subclasses features the strongest association. We show that low levels of galactosylation and sialylation and high levels of fucosylation correspond with a state of low-grade inflammation and a detrimental lipid profile, characterized by low HDLC and high TG. This is likely due to the fact that a chronic level of inflammation is a known risk factor for cardiovascular disease^79^. This high risk profile is also characteristic for ageing – in older individuals, galactosylation and sialylation decrease, while bisection increases (Supplemental Figure 2).

IgG glycosylation is known to be directly involved in the interaction with Fcγ receptors and complement, modulating the inflammatory capacity of the antibody^3,80^, whereas CRP influences opsonisation of pathogens and complement activation^81^. Considering the complexity of the immune system and the multitude of pathways therein, these regulatory mechanisms might well be (partially) additive rather than overlapping. Furthermore, the complexity of IgG glycosylation with its multiple features has the advantage that it could provide more information than the level of a single analyte such as CRP, as shown by the IgG glycosylation features exhibiting different associations with metabolic markers. For these reasons, subclass-specific glycosylation of IgG, independently or in combination with the traditional inflammation marker CRP, might be a more informative biomarker regarding inflammation and metabolic health than CRP on its own. Further studies are required to review the added value of IgG glycosylation as a biomarker in light of prospective data on cardiovascular incidence and morbidity.

## Electronic supplementary material

Supplemental Data

Supplemental Table S1

Supplemental Table S3
