# Supplementary material for: Subclass-specific IgG glycosylation is associated with markers of inflammation and metabolic health
Source: Sci Rep. 2017 Sep 26;7:12325. doi: 10.1038/s41598-017-12495-0 (PMC5615071; doi:10.1038/s41598-017-12495-0)
Supplement: Supplementary file 1 — Supplemental Data [file 41598_2017_12495_MOESM1_ESM.doc]

**Supplemental Data**

**Manuscript: Subclass-specific IgG glycosylation is associated with markers of inflammation and metabolic health**

Rosina Plomp^1^, L. Renee Ruhaak^1,2^, Hae-Won Uh^3^, Karli R. Reiding^1^, Maurice Selman^1,5^, Jeanine J. Houwing-Duistermaat^4^, P. Eline Slagboom^4^, Marian Beekman^4^, Manfred Wuhrer^1,*^

^1^ Center for Proteomics and Metabolomics, Leiden University Medical Center, Leiden, The Netherlands;

^2^ Department of Clinical Chemistry and Laboratory Medicine, Leiden University Medical Center, Leiden, The Netherlands;

^3^ Department of Medical Statistics and Bioinformatics, Leiden University Medical Center, Leiden, The Netherlands;

^4^ Department of Molecular Epidemiology, Leiden University Medical Center, Leiden, The Netherlands;

^5^ Present address: Pharming Group N.V., Leiden, The Netherlands

* To whom correspondence should be addressed:

Prof. Manfred Wuhrer

Leiden University Medical Center

Center for Proteomics and Metabolomics

Postbus 9600, 2300 RC Leiden

The Netherlands

Tel: +31-20-5987527

Fax: +31-71-5266907

m.wuhrer@lumc.nl

**
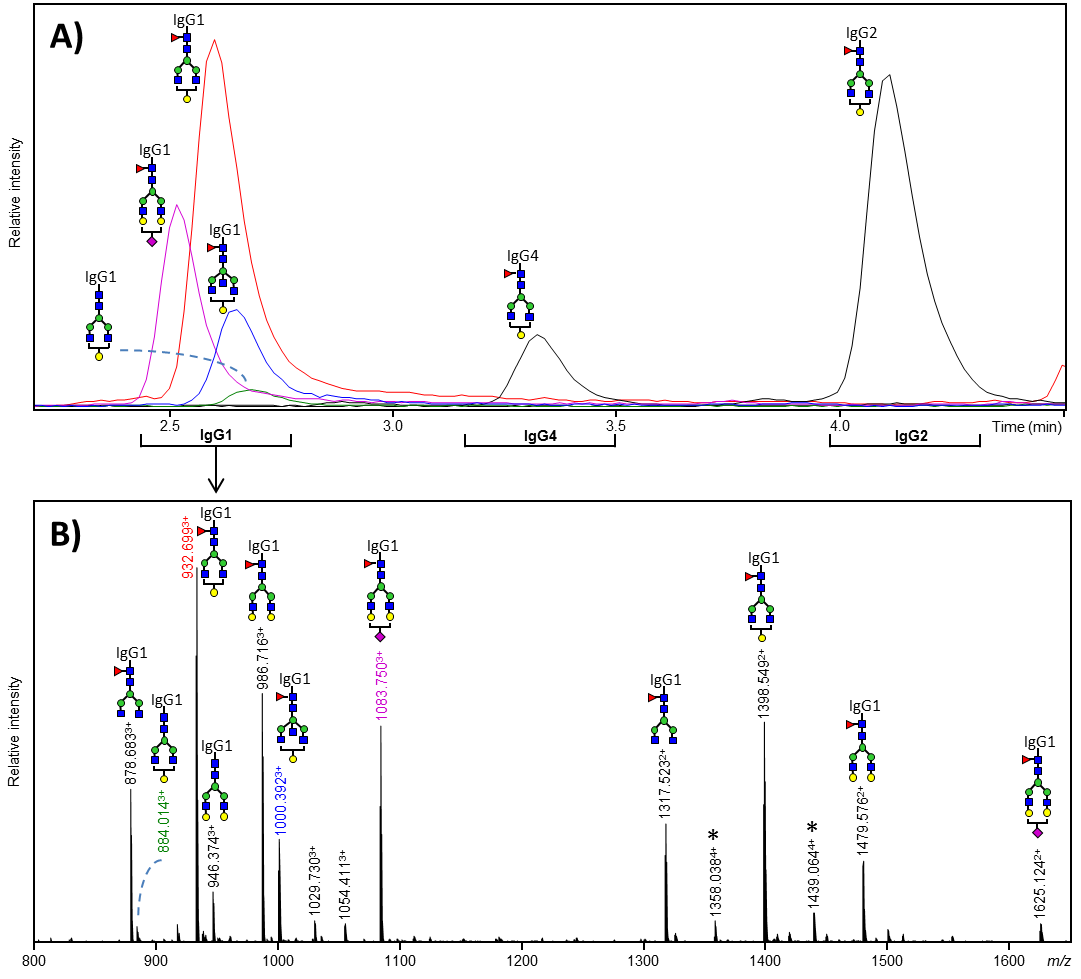
**

**Supplemental Figure S1.** A) Extracted ion chromatograms (EICs) of various IgG glycopeptides, showing that IgG1 glycopeptides elute first, followed by IgG4 and lastly IgG2. B) A mass spectrum of IgG1 glycopeptides in both 2+ and 3+ charge state. The peaks marked with an asterisk are quadruply charged dimers of two IgG1 glycopeptides.

**Supplemental Table S1.** Comprehensive list of all *N*-glycopeptides examined in the data, with the *m/z* value of the first three isotopic peaks in both 2+ and 3+ charge state. The average retention time for all *N*-glycans attached to an IgG subclass is listed.

See separate Excel file.

**Supplemental Table S2. Calculation of glycosylation features from individual *N*-glycan percentages.** The difference between glycosylation feature calculations for different IgG subclasses arises from the exclusion of IgG2 *N*-glycopeptides G1FNS1 and G2FNS1 and afucosylated IgG4 *N*-glycopeptides. F=core fucose, N=bisecting *N*-acetylglucosamine, G=galactose, S=*N*-acetylneuraminic (sialic) acid.

| **glycosylation feature** | **description** | **calculation** |
| --- | --- | --- |
| IgG1 fucosylation | % of IgG1 *N*-glycans carrying a core fucose | G0F + G1F + G2F + G0FN + G1FN + G2FN + G1FS1 + G2FS1 + G1FNS1 + G2FNS1 |
| IgG1 bisection | % of IgG1 *N*-glycans carrying a bisecting GlcNAc | G0FN + G1FN + G2FN + G1FNS1 + G2FNS1 + G0N + G1N + G2N + G1NS1 + G2NS1 |
| IgG1 galactosylation | % of IgG1 *N*-glycan antennae carrying a galactose | (G1F + G1FN + G1FS1 + G1FNS1 + G1 + G1N + G1S1 + G1NS1)*0.5 + (G2F + G2FN + G2FS1 + G2FNS1 + G2 + G2N + G2S1 + G2NS1)*1 |
| IgG1 sialylation | % of IgG1 *N*-glycan antennae carrying a sialic acid | (G1FS1 + G2FS1 + G1FNS1 + G2FNS1 + G1S1 + G2S1 + G1NS1 + G2NS1)*0.5 |
| IgG1 sialic acid per galactose | % of galactoses carrying a sialic acid on IgG1 | (IgG1 sialylation/IgG1 galactosylation) * 100 |
| IgG2 fucosylation | % of IgG2 *N*-glycans carrying a core fucose | G0F + G1F + G2F + G0FN + G1FN + G2FN + G1FS1 + G2FS1 |
| IgG2 bisection | % of IgG2 *N*-glycans carrying a bisecting GlcNAc | G0FN + G1FN + G2FN + G0N + G1N + G2N + G1NS1 + G2NS1 |
| IgG2 galactosylation | % of IgG2 *N*-glycan antennae carrying a galactose | (G1F + G1FN + G1FS1 + G1 + G1N + G1S1 + G1NS1)*0.5 + (G2F + G2FN + G2FS1 + G2 + G2N + G2S1 + G2NS1)*1 |
| IgG2 sialylation | % of IgG2 *N*-glycan antennae carrying a sialic acid | (G1FS1 + G2FS1 + G1S1 + G2S1 + G1NS1 + G2NS1)*0.5 |
| IgG2 sialic acid per galactose | % of galactoses carrying a sialic acid on IgG2 | (IgG2 sialylation/IgG2 galactosylation) * 100 |
| IgG4 bisection | % of IgG4 *N*-glycans carrying a bisecting GlcNAc | G0FN + G1FN + G2FN + G1FNS1 + G2FNS1 |
| IgG4 galactosylation | % of IgG4 *N*-glycan antennae carrying a galactose | (G1F + G1FN + G1FS1 + G1FNS1)*0.5 + (G2F + G2FN + G2FS1 + G2FNS1)*1 |
| IgG4 sialylation | % of IgG4 *N*-glycan antennae carrying a sialic acid | (G1FS1 + G2FS1 + G1FNS1 + G2FNS1 + G1S1 + G2S1 + G1NS1 + G2NS1)*0.5 |
| IgG4 sialic acid per galactose | % of galactoses carrying a sialic acid on IgG4 | (IgG4 sialylation/IgG4 galactosylation) * 100 |

**
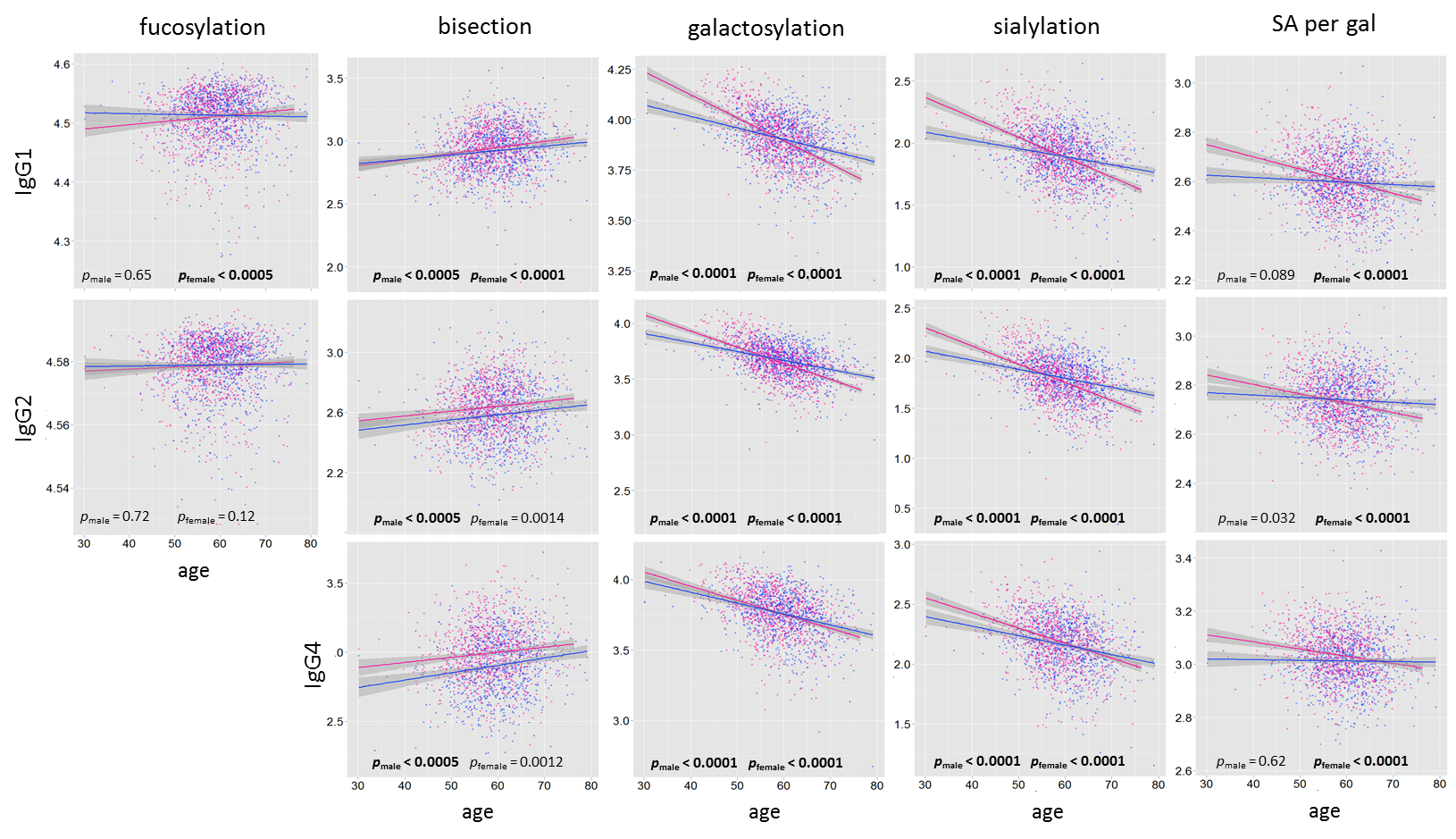
**

**Supplemental Figure S2. Age- and sex-dependence of IgG glycosylation features (male: blue, female: pink).** A trend line (linear fit) is shown with a 95% confidence interval. *P*-values for association with age were calculated separately for males and females using linear regression based on the model ‘glycosylation ~ age’. *P*-values below the Bonferroni-corrected threshold of 7.14 x 10^-4^ are shown in bold. The glycosylation features are log transformed.

**Supplemental Table S3. *P_1_*-values, β_1_ coefficients and t-statistics (=β_1_/SE(**β_1_**)) relating to associations between glycosylation features and metabolic parameters**. Correlations with a *p****_1_***-value below 7.14 x 10^-4^, which are significant after Bonferroni correction, are shown in bold. The N refers to the number of values available for each metabolic parameter.

See separate Excel file.

**Supplemental Table S4. P_1_-values, β_1_ coefficients and t-statistics (=β_1_/SE(β_1_)) relating to associations between longevity and IgG glycosylation features.** Correlations are shown for all of the study population (all), for the younger half (<60) and the older half (>60). The N refers to the number of values available for each group.

| ***p_1_*-value** | | | |
| --- | --- | --- | --- |
|  | **all** | **<60** | **>60** |
| **N** | 1826 | 1003 | 822 |
| **IgG1 fucosylation** | 0.0787 | 0.1004 | 0.2412 |
| **IgG2 fucosylation** | 0.0514 | 0.0599 | 0.2948 |
| **IgG1 bisection** | 0.4320 | 0.0524 | 0.6338 |
| **IgG2 bisection** | 0.3861 | 0.0760 | 0.5941 |
| **IgG4 bisection** | 0.1401 | 0.0561 | 0.7677 |
| **IgG1 galactosylation** | 0.8987 | 0.4848 | 0.6048 |
| **IgG2 galactosylation** | 0.9056 | 0.5370 | 0.6028 |
| **IgG4 galactosylation** | 0.0217 | 0.1051 | 0.0871 |
| **IgG1 sialylation** | 0.7284 | 0.3600 | 0.1256 |
| **IgG2 sialylation** | 0.9132 | 0.4462 | 0.3344 |
| **IgG4 sialylation** | 0.2440 | 0.1155 | 0.8207 |
| **IgG1 SA per gal** | 0.5961 | 0.4478 | 0.0789 |
| **IgG2 SA per gal** | 0.6786 | 0.6664 | 0.2644 |
| **IgG4 SA per gal** | 0.2631 | 0.4482 | 0.0274 |
| **β_1_ coefficient** | | | |
|  | **all** | **<60** | **>60** |
| **N** | 1826 | 1003 | 822 |
| **IgG1 fucosylation** | -0.10 | -0.14 | -0.09 |
| **IgG2 fucosylation** | -0.11 | -0.15 | -0.08 |
| **IgG1 bisection** | -0.05 | -0.16 | 0.04 |
| **IgG2 bisection** | -0.05 | -0.15 | 0.04 |
| **IgG4 bisection** | -0.09 | -0.15 | -0.02 |
| **IgG1 galactosylation** | 0.01 | 0.06 | -0.04 |
| **IgG2 galactosylation** | 0.01 | 0.05 | -0.04 |
| **IgG4 galactosylation** | 0.14 | 0.13 | 0.14 |
| **IgG1 sialylation** | -0.02 | 0.08 | -0.12 |
| **IgG2 sialylation** | -0.01 | 0.06 | -0.08 |
| **IgG4 sialylation** | 0.07 | 0.13 | 0.02 |
| **IgG1 SA per gal** | -0.03 | 0.06 | -0.13 |
| **IgG2 SA per gal** | -0.02 | 0.03 | -0.09 |
| **IgG4 SA per gal** | -0.07 | 0.06 | -0.18 |
| **t_1_ statistic** | | | |
|  | **all** | **<60** | **>60** |
| **N** | 1826 | 1003 | 822 |
| **IgG1 fucosylation** | -1.76 | -1.64 | -1.17 |
| **IgG2 fucosylation** | -1.95 | -1.88 | -1.05 |
| **IgG1 bisection** | -0.79 | -1.94 | 0.48 |
| **IgG2 bisection** | -0.87 | -1.77 | 0.53 |
| **IgG4 bisection** | -1.48 | -1.91 | -0.30 |
| **IgG1 galactosylation** | 0.13 | 0.70 | -0.52 |
| **IgG2 galactosylation** | 0.12 | 0.62 | -0.52 |
| **IgG4 galactosylation** | 2.30 | 1.62 | 1.71 |
| **IgG1 sialylation** | -0.35 | 0.92 | -1.53 |
| **IgG2 sialylation** | -0.11 | 0.76 | -0.97 |
| **IgG4 sialylation** | 1.16 | 1.57 | 0.23 |
| **IgG1 SA per gal** | -0.53 | 0.76 | -1.76 |
| **IgG2 SA per gal** | -0.41 | 0.43 | -1.12 |
| **IgG4 SA per gal** | -1.12 | 0.76 | -2.21 |
